# Lineage Tracing and Molecular Real-Time Imaging of Cancer Stem Cells

**DOI:** 10.3390/bios12090703

**Published:** 2022-09-01

**Authors:** Xiaohua Jia, Guodong Shen, Jia Jia, Yan Zhang, Dan Zhang, Wanjun Li, Jianjun Zhang, Xinglu Huang, Jie Tian

**Affiliations:** 1 Key Laboratory of Molecular Imaging of Chinese Academy of Sciences, Institute of Automation, Chinese Academy of Sciences, Beijing 100190, China; 2 Department of Ultrasound, General Hospital of People’s Liberation Army, Beijing 100853, China; 3 Department of General Surgery & Guangdong Provincial Key Laboratory of Precision Medicine for Gastrointestinal Tumor, Nanfang Hospital, Southern Medical University, Guangzhou 510515, China; 4 Fenyang College, Shanxi Medical University, Fenyang 032200, China; 5 Center of Biomedical Analysis, Tsinghua University, Beijing 100084, China; 6 Department of Pathology, Affiliated 3201 Hospital of Xi’an Jiaotong University, Hanzhong 723000, China; 7 Key Laboratory of Mental Health, Institute of Psychology, Chinese Academy of Sciences, Beijing 100101, China; 8 Key Laboratory of Bioactive Materials for the Ministry of Education, College of Life Sciences, State Key Laboratory of Medicinal Chemical Biology, Nankai University, Tianjin 300071, China; 9 Beijing Advanced Innovation Center for Big Data-Based Precision Medicine, School of Medicine and Engineering, Beihang University, Beijing 100191, China

**Keywords:** lineage tracing, molecular imaging, cancer stem cells

## Abstract

The cancer stem cells (CSC) are the roots of cancer. The CSC hypothesis may provide a model to explain the tumor cell heterogeneity. Understand the biological mechanism of CSC will help the early detection and cure of cancer. The discovery of the dynamic changes in CSC will be possible by the using of bio-engineering techniques-lineage tracing. However, it is difficult to obtain real-time, continuous, and dynamic live-imaging information using the traditional approaches that take snapshots of time points from different animals. The goal of molecular imaging is to monitor the in situ, continuous molecular changes of cells in vivo. Therefore, the most advanced bioengineering lineage tracing approach, while using a variety of molecular detection methods, will maximize the presentation of CSC. In this review, we first introduce the method of lineage tracing, and then introduce the various components of molecular images to dynamic detect the CSC. Finally, we analyze the current situation and look forward the future of CSC detection.

## 1. Introduction

Cancer stem cells (CSC), or tumor-initiating cells, are a unipotent cell population presented within the tumor cell mass, with the ability to self-renew and differentiate and to drive the growth and metastasis of tumors [1]. The CSC model provides one explanation for the phenotypic and functional heterogeneity among cancer cells. CSC may arise from dysregulated transformation of normal stem cells and progenitor cells. CSC eradication is critical from a clinical perspective. The development of novel approaches to target CSC will be a promising diagnosis and therapeutic strategy.

Lineage tracing is the identification of all progenies of a single CSC, which is an important bio-engineering approach to examine the self-renewal and differentiate of CSC in vivo. A previous study showed that a genetic marker for healthy intestinal stem cells was also expressed in benign intestinal cancer stem cells, which are precancerous lesions of the tumor [2]. In other study, mice were designed to carry genes for drug-induced markers that, when activated, caused the labeled cells to produce one of four types of fluorescence. This experiment produced a monochromatic tumor composed of several cell types, indicating that each tumor was from a single stem cell [3]. Therefore, it is necessary to find out such CSC that are likely to become tumors.

However, the in situ, continuous imaging of deep CSC is difficult. Traditional lineage tracing results are mainly presented by microscopic snapshots at different time points. The occurrence and development of tumors is a process in which multiple molecules change over time. Therefore, close monitoring of temporal and spatial changes of each marker is necessary to predict possible progression and early detection. The definition of molecular imaging method is to dynamically monitor the molecular changes in vivo. Combining advances in both lineage tracing and imaging techniques will allow real-time dynamic analysis of CSC. Here, we review the most advanced bioengineering lineage tracing approach, while using a variety of detection methods, hoping to maximize the presentation of CSC. We first introduce the method of lineage tracing, and then introduce the components of various molecular imaging. Finally, we analyze the current situation and look forward to the future of detection.

**Figure 1:**
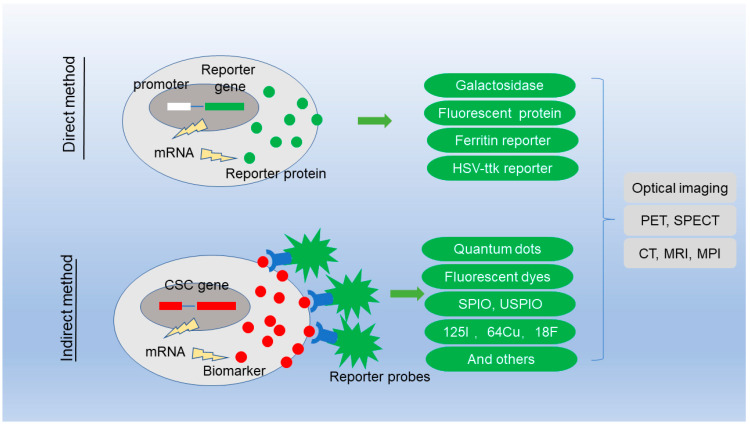
Imaging strategies for the detection of cancer stem cells.

The detection of CSC has always been the focus of the research controversy. The lineage tracing is achieved through a direct genetic modification method. Individual cells are labeled in such a way that the tag is delivered to the progeny of the cells, producing a set of labeled clones. Lineage tracing by direct observation is not possible in clinical situation, and indirect molecular imaging method has been developed to label the CSC. Molecular imaging is generally defined as noninvasive imaging of cellular and molecular events, which quantified physiological changes using imaging probes or beacons. Figure 1 shows the two imaging strategies to detect CSC.

## 2. Lineage Tracing

Lineage tracing is a powerful method to delineate all progenies produced by a single cell, which can be achieved by site-specific recombinase (SSR) technology. SSR technology was pioneered in the 1980s [4]. Through a recombinase-mediated DNA breakage and joining process, SSR can induce the DNA excision, integration, resolution, or inversion of target genes. Three popular tools used in genomic engineering are FLP/FRT from the 2-μm plasmid of *Saccharomyces cerevisiae*, Cre/loxP from the *Escherichia coli* phage P1, and phiC31/*att* from the *Streptomyces* phage phiC31. The Cre/loxp system has higher recombination efficiency than the other strategies.

### 2.1. Inducible Cre/Loxp

The Cre/loxP system is a widely used tool for lineage tracing studies in mice. The Cre recombinases can mediate specific recombination of gene sequences riveted by loxp. The Cre/loxp system was first used in vivo cell lineage tracing studies by Joyner et al. [5]. They constructed two kinds of transgenic mice in the process of studying brain development. The marker gene LacZ was inserted after the promoter of the β-actin gene in one mouse. The expression of the marker gene was disturbed by a loxP flanked stop sequences. In another mouse, the Cre gene was inserted after the Engrailed2 (En2) promoter. In the progeny of these two transgenic mice, cells expressing En2 produced the enzyme Cre, and then the stop sequence was removed, and the marker gene LacZ can be successfully expressed. Using this method, Joyner et al. demonstrated the important role of En2-positive cells in mouse midbrain development. After that, Soriano et al. constructed a gene-directed knock-in mouse with the LacZ reporter gene inserted at the ROSA26 site [6]. The mouse was based on the ROSAβgeo26 gene trap (Gene-trap) mouse strain, and the upstream insertion of the LacZ gene was loxp flanked stop sequence, indicating Cre expression. The knock-in gene expresses more efficiently and does not interfere with the normal expression of other genes.

Compared with the other strategies, the recombination efficiency and targeting ability of Cre/loxp system is high enough for an easy recovery of rearranged clones without any selection system. In addition, the greatest advantage of the Cre/loxp system is that the genome has been modified and genetically characterized, and the cells expressing Cre during development and all the cells of their descendants will be permanently marked. However, one disadvantage of this system is the inability to control the marking time.

To solve the problem, Metzger et al. made a new Cre/loxp mouse model [7]. The new model used estrogen receptors to regulate the timing of Cre entry in the nucleus, which are nuclear receptors. Cre in the model was expressed fused to a human estrogen receptor ligand-binding domain (Cre-ERT). The estrogen receptors have affinity for tamoxifen and do not bind estradiol. When animals are not injected with tamoxifen, Cre cannot enter the nucleus because estrogen receptors are still present in the cytoplasm. Only under the condition of exogenous tamoxifen can Cre enter the nucleus with the estrogen receptor to realize the recombinant modification of the loxp fragment. Therefore, the temporal control of the knockout event can be achieved, just through controlling the injection timing of tamoxifen into the body. Barker et al. used Lgr5-EGFP-creERT2 knock-in mice and ROSA26-LacZ mice for mating [8]. Their progeny was injected with tamoxifen for a certain time, so that the cells expressing lgr5 during this period were permanently marked by LacZ. By imaging labeled cells, it was found that the LacZ expressed cells would gradually differentiate into various types of intestinal epithelial cells within 60 days. The results proved that the Lgr5 gene was specifically expressed in intestinal epithelial stem cells, which suggested that it was an intestinal stem cell gene marker.

### 2.2. Multicolor Reporter Constructs

Compared with single-labeled gene reporter mice, the compound labeling method can better indicate the location of target cells. The earliest compound-labeled mice included Z/AP and Z/EG, both of which were based on the Cre/loxp system. Z/AP mice expressed the lacZ gene prior to Cre-mediated excision. After Cre-mediated excision of the lacZ gene, Z/AP mice expressed the human alkaline phosphatase gene (ALP), which was the second reporter gene [9]. The limitations of Z/AP mice were that positive cells cannot be detected by flow cytometry, and both markers can only be visualized by section staining. The principle of Z/EG and Z/AP mouse was similar, which was composed of LacZ and EGFP. The advantage of Z/EG was being able to observe EGFP fluorescence in living tissue, making it easier to track labeled cells [10]. The dual fluorescent protein mT/mG mouse was another compound-labeled mouse. After Cre-mediated recombination modification, the mT/mG mouse was converted from expressing tdTomato to EGFP. The fluorescent proteins tdTomato and EGFP were both expressed on the cell membrane. Under fluorescent excitation conditions, labeled cells in mT/mG can be visualized. Labeling of fluorescent proteins greatly improved the resolution of lineage tracing observations. Bowman et al. used Axin2-CreERT2 mice and Rosa26-mT/mG mice, showing that the Wnt/β-catenin pathway played an important role in maintaining homeostasis and function, during neural stem cell development [11]. The multi-fluorescent labeling system Brainbow was a composite labeling system applied to zebrafish, which enabled different cells in vivo to display fluorescent signals of different colors. Livet et al. found that in the Brainbow system, the multiple fluorescent colors of various cells could be stably inherited with the passage of cells [12]. 

### 2.3. CRISPR-Cas9 Genome-Editing Technology

Single-cell lineage tracing technologies based on CRISPR–Cas9 technology have been used to track of cell lineages in diverse cells, tissues, and lower vertebrates [13]. CRISPR ‘barcodes’ mammalian development in Genome editing technology allows researchers to trace cell lineages in developing mice. McKenna et al. developed synthetic arrays of 9 to 12 CRISPR/Cas9 target sites to generate thousands of unique derivative barcodes for whole-organism lineage tracing [14]. This approach enabled cell lineage tracing by evolving genetic barcoding technology, recorded the process of cell division in developing mice, and traced the realization of each lineage. 

## 3. The Promoter or Marker of Cancer Stem Cell

### 3.1. The Promoter of CSC

Some studies have suggested that the presence of specific marker-based cancer progenitor cell populations in tissues. The lineage tracing is achieved through a direct genetic modification method. Individual cells are labeled in such a way that the tag is delivered to the progeny of the cells, producing a set of labeled clones.

Parada and his colleagues tested whether Nestin could label glioma CSC. Previously, only adult neural stem cells could express Nestin, and in their directionally differentiated offspring there was no Nestin expression [15]. Through the using of stereotactic delivery of Nestin-cre-ERT2 transgene to the SVZ, they described the temporal and spatial limitations of gene targeting of brain neurogenic niches in vivo. They found that all tumors contained many unlabeled cells, and at least a few labelled cells. Further experiments showed that unlabeled cells originated from the labeled precursors. The Nestin labelled cells were possibly CSC. 

Research reports in 2016 show that Lgr6 can mark a rare mammary gland cell population capable of producing luminal breast tumor [16]. To test whether Lgr6^+^ cells can act as mammary tumor-initiating cells, the researchers inactivated the two most common mutant breast tumor suppressors, Brca1 and Trp53, in Lgr6^+^ cells. Lgr6^+^ cells expressed the luminal markers Era and K8 and formed breast tumors. The results demonstrated that oncogenic mutations in Lgr6^+^ cells could lead to luminal carcinoma. Furthermore, they activated tumorigenic K-RasG12D, and deleted the tumor suppressor WD and F-box repeat domain, in Lgr6^+^ cells to assess the tumor-initiating capacity of cells in a second tumor model. Tumors in such animals developed rapidly. Luminal lesions appeared after 3 weeks of tamoxifen use. Breast cancer expressed K8 and Era after 8 weeks of tamoxifen. In both breast cancer models, 100% of Lgr6^+^ cell-derived tumor cells stained positive for K8. Lgr6^+^ cells were therefore potent tumor-initiating cells for luminal mammary tumors.

There were CSC in the tumor, and CSC were responsible for the heterogeneity of the tumor. The markers of these tumor progenitor cells showed during cancer progression, combined with imaging equipment through the construction of lineage tracking (Table 1).

### 3.2. The Biomarker of CSC

Cancer treatment is evolving towards personalized precision therapy. CSC biomarkers have become an increasingly important part of cancer diagnosis and treatment because they can be used to reveal disease status or predict disease progression. The detection of CSC biomarkers in clinical work usually relies on the detection of isolated body fluids or tissues. Clinical trials investigating the role of CSC in patients have been hampered by the lack of non-invasive, real-time, and quantitative CSC biomarker technologies.

CD133/prominin-1/AC133 is a pentaspan transmembrane glycoprotein. CD133 positive CSC existed in many types of tumors, including glioblastoma multiforme, lung, liver, colon, pancreatic, and melanoma, ovarian cancer, leukemia, and sarcomas. Simone Gaedicke used a radiolabeled CD133-specific antibody, and successful detected the CSC in mice by noninvasive positron emission tomography (PET) [17]. CD146 is a promising target for therapy of many different carcinomas and noninvasive imaging in vivo. Yunan Yang developed a ^64^Cu-radiolabeled anti-CD146 antibody (YY146). ^64^Cu-YY146 can be used to specifically detect the orthotopic and subcutaneous brain tumors using PET. In addition, YY146 can detect CD146^+^ CSC in other tumor tissues, such as ovary, lung, liver, and stomach [18].

**Table 1:** Lineage tracing of CSC.

Tumor Type	Gene Marker	Report Gene	Imaging	Year
Acute lymphoblastic leukemia	Lmo2	TdTomato	optical imaging	2018 [19]
Breast cancer	CD44	luciferase	optical imaging	2010 [20]
	Lgr6	GFP, RFP, tdTomato	optical imaging	2016 [16]
	PIK3CA	GFP, RFP, tdTomato	optical imaging	2015 [21]
	SOX9	tdTomato	optical imaging	2017 [22]
	Gpr125	GFP, tdTomato	optical imaging	2022 [23]
Brain cancer	TlX	GFP	optical imaging	2014 [24]
Medulloblastoma	Sox2	GFP, tdTomato	optical imaging	2020 [25]
Head and neck cancer	BM1	GFP, RFP, tdTomato	optical imaging	2017 [26]
	CD276	tdTomato	optical imaging	2021 [27]
Colon cancer	LGR5	GFP, RFP, tdTomato	optical imaging	2017 [28]
	LGR5	GFP, RFP, tdTomato	optical imaging	2012 [29]
	Prom1	GFP, RFP, tdTomato	optical imaging	2009 [30]
	DCLK1	GFP, RFP, tdTomato	optical imaging	2014 [31]
	DCLK1	GFP, RFP, tdTomato	optical imaging	2013 [32]
	IL17RB	GFP, tdTomato	optical imaging	2019 [33]
Liver cancer	EpCAM	tdTomato, LacZ	optical imaging	2017 [34]
	Prom1	tdTomato	optical imaging	2021 [35]
Lung cancer	AT2	GFP, RFP, tdTomato	optical imaging	2014 [36]
Pancreatic cancer	Musashi	YFP	optical imaging	2016 [37]
	DCLK1	GFP, RFP, tdTomato	optical imaging	2014 [38]
	DCLK1	GFP	optical imaging	2021 [39]
Prostate cancer	BM1	GFP, RFP, tdTomato	optical imaging	2016 [40]
	Pten	GFP, RFP, tdTomato	optical imaging	2014 [41]
	RUNX1	GFP, RFP	optical imaging	2020 [42]
	LY6D	YFP	optical imaging	2018 [43]
gastric cancer	eR1	GFP, tdTomato	optical imaging	2017 [44]
	LGR5	GFP, tdTomato	optical imaging	2017 [45]
	LGR5	GFP, tdTomato	optical imaging	2015 [46]
	CCK2R	GFP, tdTomato	optical imaging	2015 [47]
	LGR5	GFP, tdTomato	optical imaging	2015 [48]
Bladder cancer	K5	GFP, tdTomato	optical imaging	2014 [49]
Endometrial cancer	Axin2	GFP, YFP, LacZ	optical imaging	2020 [50]

The targeted molecular imaging enabled specific biomarker of CSC to be detected (Table 2). Although the hypothesis that CSC contribute to the development of cancer is attractive and partially confirmed by preclinical and some clinical studies, successful clinical non-invasive, real-time imaging using ligands such as antibodies, peptides, and small molecules is rare.

## 4. Reporter Gene and Probes

### 4.1. Reporter Gene

Both direct and indirect imaging require substances that can be used for imaging. Lineage tracing uses proteins that can be expressed by genes. Compared with the indirect labeling method, the direct labeling method using reporter genes has many advantages. Stable transfection of the reporter gene in cells can ensure its long-term expression in dividing and proliferating progeny cells. Expression of the reporter gene also confirms that the tagged cells are viable. Indirect labeling methods rely on probes. Because the probe is diluted in progeny cells, the labeling is not stable, and the signal of the probe is not correlated with the survival of progeny cells.

#### 4.1.1. Galactosidase

β–galactosidase was one of the first reporters exploited for lineage tracing, which was encoded by extensively used *Escherichia coli* lacZ gene. When incubated with the substrate analogue X–gal, β–galactosidase produced an intense blue precipitate. Alternatively, β–galactosidase can be visualized by an immunofluorescent staining technique.

#### 4.1.2. Fluorescent Reporters

The earliest fluorescent proteins were found in *Aequorea victoria* by Shimomura et al. (1962) [59], and more than 30 fluorescent proteins with different wavelengths were subsequently discovered, including green fluorescent protein (GFP), yellow fluorescent protein (YFP), red fluorescent protein (RFP), and orange fluorescent protein. Because of its strong fluorescence signal, fluorescent protein is easy to observe and track. It has been widely used in tracking research. The commonly used fluorescent protein is GFP. Although its fluorescence intensity is slightly weaker than that of RFP, it is the least toxic to cells. In addition, tdTomato is also a commonly used fluorescent protein, which is a variant of RFP, has a stronger fluorescence intensity than green fluorescent protein, and can be quickly degraded into monomers to reduce cytotoxicity. The dual-fluorescent membrane Tomato/membrane Green (mT/mG) mouse constructed by the Cre/loxp site-specific recombination system is based on the characteristics of fluorescent proteins, which can change the fluorescence color under the action of Cre, thereby improving the resolution of cells in tissues.

#### 4.1.3. MRI Reporter Gene

The reporter gene for MRI is mainly ferritin. Cells overexpressing ferritin absorb more iron and show low signal intensity on MRI. Choi et al. used a dual reporter gene for enhanced green fluorescent protein (EGFP) and ferritin to monitor the in vivo behavior of transplanted human breast CSC in mice using MRI [60]. MRI showed that the signal intensity of ferritin gene-tagged CSCs and control cells was significantly different in vitro and in vivo. Ferritin-based MRI signals, compared to EGFP fluorescence signals, had higher spatial resolution, and were not affected by imaging depth. MRI reporter gene markers can be used as a reliable method to identify CSC-derived progeny cells and may serve as a new tool to dynamically monitor the efficacy of drug-targeted CSC therapy in vivo.

#### 4.1.4. PET Reporter Gene

The principle of radionuclide reporter gene imaging is to genetically engineer cells to express receptors, enzyme, or transporters of radioactive probes to promote the uptake and aggregation of radioactive probes by target cells. PET or SPECT detects tracers with bioemission photon activity. Relying on the labeling of cells with tracers, imaging devices enable in vivo monitoring of cells. Wild-type herpes simplex type 1 thymidine kinase (HSV1-ttk) and its HSV1-sr39tk mutant genes are the most widely used enzyme reporter genes in PET molecular imaging studies. Thymidine kinase negatively charges the cell surface by phosphorylating the radionuclide matrix, preventing the radiolabel from detaching from the cell. HSV1-ttk has been used with radioactive probes such as FIAU, FEAU and FHBG. Radionuclide reporter gene imaging technology has some shortcomings. First, continuous injection of radionuclides into organisms can cause potential radioactive damage to the organism. In addition, the short half-lives of most currently available radiotracers limit this imaging method to short-term acute biodistribution imaging. Due to the immune response caused by the HSV-ttk viral protein in the human body, the translation of the HSV-ttk reporter gene into the clinic is restricted. 

### 4.2. Targeted Nanoparticles

Direct detection of reporter genes is superior to indirect detection methods of nanoparticles in terms of precision and accuracy. Since direct detection is not clinically applicable, indirect methods of imaging cell clones in living tissue with various nanoparticles are more likely to translate to the clinic. Indirect detection methods are achieved through the specific binding of imaging contrast agents to CSC biomarkers. To achieve the imaging goal of indirect detection, contrast materials such as fluorophores appropriately functionalized on the surface with receptors, ligands, or small molecule oligomers are used to identify specific biomarkers of CSC.

#### 4.2.1. Fluorescence Probes

Fluorescent probes have the advantages of high sensitivity, strong selectivity, and versatility. These excellent properties make fluorescent probes one of the most useful tools in molecular imaging research. Fluorescent probes include dyes, Quantum Dots, AIE, and other optical nanoparticles. Compared with traditional fluorescent organic dyes, quantum dots have higher photostability, stronger fluorescence, and other properties. An integrative strategy has been developed to produce CD44-targeted NIR-sensitive hydrogels (Cy5.5-conjugated hyaluronic acid/polyethyleneimine polyplexes) for the precise identification of CD44 positive gastric CSC [61]. The probe had good biocompatibility, molecular imaging potential, and can be used for specific targeting of biomarkers and non-invasive imaging.

#### 4.2.2. MRI Probes

MRI is a non-ionizing imaging technique. Through detecting the nuclear spins of hydrogen atoms in the human body, which are mainly derived from water and fat, MRI produces good signal contrast in soft tissues. By respectively accelerating the T1 or T2 relaxation of water protons, MRI contrast material can provide bright or dark contrast signals. Superparamagnetic iron oxide (SPIO) or ultrasmall super paramagnetic iron oxide (USPIO) are traditional magnetic resonance probes. Magnetic nanoparticles, including manganese and gadolinium nanoparticles, can also be used for MR images. Advantages of these contrast agents are higher contrast enhancement, sub-nanomolar-range detection limits, and low toxicity. For example, SPIO has successfully detected and tracked glioblastoma CSC in vitro and transplanted human hepatic stem cells in vivo.

#### 4.2.3. PET Probes

Positron emission tomography (PET) is a noninvasive molecular imaging modality. PET allows noninvasive quantitative assessment of biochemical and functional processes. Radioactive bioactive molecules such as ^125^I, ^64^Cu, ^68^Ga, and ^18^F were used for PET imaging. Chen et al. used ^125^I-labeled ANC9C5, an anti-human CD133 antibody to investigate the in vivo radionuclide imaging potential of CSC in colon carcinoma xenografts [18]. The results showed that CD133 immunohistochemistry expression was overlapped with intra-tumoral distribution of ^125^I-labeled ANC9C5 depicted on autoradiography in many areas. Gaedicke et al. used ^64^Cu-NOTA-CD133 to perform PET imaging.

## 5. Molecular Imaging Technologies

Advances in imaging techniques to visualize cellular events are expected to lead researchers to a deeper understanding of CSC. Modalities used for in vivo imaging of CSC include optical imaging, magnetic resonance imaging (MRI), positron emission tomography (PET), and so on. Each modality has virtues and flaws. The comparison of different modalities in the field of cell imaging is shown in Table 3. Some of these modalities are promising as clinical applications. In CSC imaging, we need to select specific biological targets, reporter genes or probes, space requirements, and corresponding imaging modalities. Since CSC are a rare subset of cells, the resolution of the imaging modality should be sufficient to detect very low numbers of cells. Based on that, the contrast agent is indeed sensitive to represent the target. 

### 5.1. Optical Imaging

The most common used modality in CSC research and clinical practice is optical imaging. Optical imaging techniques are characterized by high sensitivity and high resolution, are the easiest used in CSC studies at the resolution of a single cell, and are relatively inexpensive compared to other imaging modalities. Optical imaging techniques include autofluorescence imaging, excited fluorescence imaging, photoacoustic imaging, light sheet imaging, etc. Excited fluorescence imaging is currently the best option for CSC imaging. A typical excited fluorescence imaging system is mainly composed of a CCD camera, an imaging camera obscura, a laser, excitation and emission filters, a constant temperature stage, a gas anesthesia system, a computer for data acquisition, data processing software, etc. Using a high-performance cooled CCD to project the luminescence at a specific location in a living animal, the low-level fluorescence signal emitted from the internal organs of the small animal is detected. Then, the obtained projection image is superimposed with the normal image of the small animal to realize the quantification of the biofluorescence of a specific position of the small animal. Sensitive detector, the intensity and stability of the fluorescent signal enable high-resolution imaging of fluorescent cells or CSC-labeled fluorescent probes in vivo. Furthermore, using multiple fluorophores, the complex biology of CSC can be imaged. The disadvantages of optical imaging include poor penetration and phototoxicity. Therefore, when selecting an optical imaging device, the detection time point, the tissue penetration depth, and the multispectral unmixing technology need to be considered.

### 5.2. Magnetic Resonance Imaging (MRI) and Magnetic Particle Imaging (MPI)

MRI is a non-invasive and high spatial resolution imaging modality. From a clinical point of view, MRI does not rely on radioisotopes and has no imaging depth limitations. MRI can collect the morphological information of the diseased tissue and the pathophysiological information of the diseased and surrounding tissue. The rapid development of MRI, especially the development of new imaging agents and imaging technologies, enables MRI in vivo imaging to reach the cellular and subcellular levels. Magnetic resonance tracking cell imaging technology is to use MR contrast agent to label cells by endocytosis or transfection, and then perform MR imaging. Superparamagnetic iron oxide particles (SPIO) are a commonly used contrast agent. MRI detects inhomogeneities in the localized proton relaxation produced by SPIO, resulting in a specific contrast in labeled cells, tissues, and body fluids. The ability of magnetic resonance contrast agents to label cells can provide a dynamic assessment of cell migration into target tissues.

Magnetic particle imaging (MPI) is a new generation of molecular imaging technology derived from MRI. It can use a composite combined rotating variable gradient magnetic field to directly detect superparamagnetic iron oxide nanoparticles in vivo to obtain ultra-high sensitivity imaging at the nanomolar level. The MPI imaging principle is based on detecting the nonlinear magnetization response of SPIO in an external time-varying magnetic field. Today, field-free point (FFP) MPI and field-line-free MPI are two branches of two technology routes that MPI is rapidly developing, which are widely accepted. MPI has the characteristics of high temporal and spatial resolution, high sensitivity, no limitation of scanning depth, and no ionizing radiation. It has been used in preclinical fields such as cell tracking, vascular imaging, and tumor imaging and therapy.

### 5.3. Positron Emission Tomography (PET)

Compared with other imaging modalities, radionuclide imaging has higher sensitivity than MRI, and radionuclide imaging has better tissue penetration than optical imaging. Unlike other molecular imaging modalities that detect nanoparticles directly, radionuclide imaging detects radioactive labels, not nanoparticles. The distribution of nanoparticles is indirectly measured by assessing the localization and quantification of radionuclides. Therefore, the most important advantage of radionuclide imaging is the ability to quantitatively detect changes in radionuclide concentration over time, thereby giving quantitative results for CSCs. PET is independent of the depth from which the signal is emitted, and is non-invasive, highly sensitive, and permits real time tracking in vivo. However, to date, detection of CSC at single-cell resolution has not been possible with PET techniques.

## 6. Discussion

CSC are associated with tumorigenesis, treatment resistance, and tumor recurrence. Although CSC have been extensively studied, their behavior in vivo remains unclear. The fate of CSC should be followed by real-time, dynamic observation. In fact, this approach is limited by the ability to identify interesting cells at the time of initiation and to identify progeny of tumor progenitors at the appropriate time.

Direct observation has the unique advantage of observing lineage progression, as it does not disturb cells or organisms. When combined with additional reporter gene to unambiguous promoter of interesting CSC, the combination of labeling strategy and detection method promotes the direct observation of CSC. For example, compared to conventional microscopy, two-photon microscopes combined with labeling strategy gives rise to several advantages. First, penetration depth of the excitation beam is increased. Second, two-photon microscopes provide great resolution for live animals. Third, two-photon excitation markedly reduces overall photobleaching and photodamage, resulting in extended viability of biological specimens during long-term real-time imaging capabilities of tissues and organisms. This type of information acquisition is a challenge for traditional lineage tracking that obtains information from different animals by the snapshot method.

Although direct observation of CSC has a unique advantage, the technology cannot be used in clinical cancer detection. The idea of in situ and real-time watching CSC behavior and key regulatory pathways within the CSC lineage for contributing to its downstream is attractive. The development of indirect imaging modalities to localize CSC will help to observe CSC for achieving complete cure of clinical cancers. Fluorescence imaging, MRI, and PET are the most promising clinical methods for CSC detection. The discovery of better biocompatible tracers, the development of better techniques, and higher imaging resolution and contrast are under investigation. Compared with other tracer techniques, MPI has the characteristics of high sensitivity, short scanning time, no tissue depth limitation, and no radioactive and ionizing radiation. Therefore, it has certain advantages in CSC tracking. At present, the main contrast agent used in MPI imaging is exogenous SPIO. In the future, MPI imaging of CSC may be possible by using ferritin gene-tagged cells. Molecular imaging that integrates all aspects of CSC detection will accelerate the translation from the laboratory to the bedside.

Advanced imaging techniques allow CSC to track and obtain valuable information, such as tumor development from the CSC population. These techniques can be used to study unexplored behaviors, such as behavior under hypoxic conditions and interactions with the immune system. To ensure the accuracy of CSC detection, direct lineage tracing imaging of CSC can be used as a standard for preclinical indirect nanoparticles imaging testing. Meanwhile, in the future, the development of clinical molecular imaging will help to make true the patients’ molecular real-time pathology, and according to the individual differences of molecular information, individualized and precise treatment can be achieve through giving the targeted drug.

## Figures and Tables

**Table 2:** Indirect molecular imaging of CSC.

Tumor Type	Biomarker	Probes	Imaging	Year
Leukemia	CD117, CD96	ICG	optical imaging	2011 [51]
Brain cancer	CD133	^64^Cu, Alexa 680,	PET, optical imaging	2014 [17]
	CD133	USPIO	MRI	2015 [18]
	CD146	^64^Cu	PET	2015 [52]
	CD133	Gold nanoparticles (GNPs)		2017 [53]
	TfR1	IRey800	optical imaging	2020 [54]
Prostate cancer	PSCA	Au/Fe(3)O(4)	MRI	2012 [55]
gastric cancer	CD44	Manganese ferrite nanoparticles (MFNPs)	MRI	2016 [56]
Pancreatic cancer	CD326	Gadolinium ion-doped upconversion nanoparticles (UCNPs)	MRI	2018 [57]
Melanin cancer	CD44	All-trans-retinoic acid (ATRA)	optical imaging	2018 [58]

**Table 3:** The comparison of different modalities in the field of CSC imaging.

Modality	Spatial Resolution	Imaging Speed	Sensitivity	Primary Cell Tracer
Optical imaging	20 μm	s-min	μmol	Optical absorption
PET	1000~2000 μm	min	pmol	Radioactive tracers
MRI	25~500 μm	min	mmol	Water protons
MPI	250~1400 μm	s-min	0.1 μmol	SPIO

## Data Availability

Not applicable.
